# Accuracy of Cervical Pedicle Screw Placement Using a Patient-Specific Template Guide System in Revision Cervical Spine Surgery: A CT-Based Morphometric and Accuracy Analysis

**DOI:** 10.7759/cureus.107607

**Published:** 2026-04-23

**Authors:** Kesavan Ramachandran, Akira Fukushima, Hiroyuki Hasebe, Hirohito Takeuchi, Shigeki Oshima, Masanori Fujiya, Itaru Oda

**Affiliations:** 1 Spine Surgery, Hospital Sultan Ismail, Johor Bahru, MYS; 2 Spine Surgery, Hokkaido Orthopaedic Memorial Hospital, Sapporo, JPN

**Keywords:** cervical morphology, cervical pedicle screws, revision surgery, screw accuracy, template-guided systems

## Abstract

Purpose: Revision cervical spine surgery is associated with specific technical challenges associated with cervical pedicle screw (CPS) placement because of altered posterior bony anatomy, scar tissue, and limited anatomical landmarks. Although patient-specific template guide systems (TGS) have shown a high level of accuracy in primary CPS placement, there is limited evidence on their application in revision cases. This study aimed to evaluate the accuracy of placement of CPS by using TGS in revision cervical spine surgery and to analyse cervical morphometric parameters across different vertebral levels to better contextualise the feasibility of screw placement and their safety. Given the limited sample size, morphometric comparisons across vertebral levels were considered exploratory and hypothesis-generating.

Methods: This was a retrospective analysis using CT of 15 patients who underwent posterior cervical instrumentation with a patient-specific TGS after previous posterior decompression surgery. A total of 90 CPS inserted from C2 until C7 were evaluated. Screw placement accuracy was determined on postoperative CT and graded based on known criteria of deviation. Radiographic parameters of the cervical pedicle and the trajectory of the screws, such as pedicle width, pedicle transverse angle, pedicle medial offset, screw diameter, screw length, pedicle screw transverse angle, and pedicle screw medial offset, were measured. Post-hoc comparisons (Dunn-Bonferroni) were performed with the Kruskal-Wallis test to analyse the inter-level differences.

Results: The study demonstrated a high rate of optimal screw placement, with 95.6% of screws classified as Grade 0 (completely contained within the pedicle), and 4.4% showing minor (<2 mm) cortical breach (Grade 1). There were no Grade 2 or Grade 3 deviations, and there were no neurovascular problems. Pedicle width was significantly different between cervical levels (chi 2 = 19.08, p = .002), with a significantly greater pedicle width at C2 than at C3, C4, and C6 (post hoc). Significant inter-level differences were also detected for all other radiographic and screw parameters (all p < .001), with upper cervical levels showing a distinct morphometric appearance.

Conclusion: CPS placement with patient-specific TGS in revision cervical spine surgery was found to have high accuracy and clinical safety. Significant anatomical variation across cervical levels at C2 emphasizes the significance of detailed preoperative planning. These findings support the feasibility of TGS-assisted CPS placement in revision settings.

## Introduction

Cervical pedicle screw (CPS) fixation is widely recognised to offer the best biomechanical stability among posterior cervical fixation techniques and superior pull-out strength and three-column fixation compared with lateral mass screw constructs, especially in cases of deformity, trauma, tumour, or multilevel instability, and thus requires rigid posterior stabilisation [1-3]. Despite these benefits, CPS insertion is still a technically challenging procedure. Cervical pedicles are small, have pronounced inter- and intra-level anatomical variation, and are surrounded by important neurovascular structures such as the vertebral artery laterally and spinal cord and nerve roots medially, making even minor deviations potentially catastrophic [4,5]. These technical challenges are amplified in revision cervical spine surgery due to possible previous decompression, laminoplasty, or laminectomy, which may cause partial or complete loss of posterior bony landmarks and alteration of the surface anatomy and the affected reference points for determining a conventional trajectory [6,7].

Traditional freehand CPS insertion techniques have been shown to have relatively high rates of pedicle wall breach, with rates of perforation anywhere from around 6.7-25% or more, depending on surgeon experience, spinal level, and assessment criteria [4,8]. Clinically significant infringements can result in serious problems such as injury to the vertebral artery and the spinal cord and nerve roots. While intraoperative navigation techniques and robotic-assisted systems have been proven to be more accurate than freehand techniques in performing CPS, their usefulness in revision settings may be limited. Registration errors may occur when either posterior bony anatomy is distorted or lacking, which can lessen the navigational reliability and possibly negate the accuracy advantage [9,10].

Patient-specific template guide systems (TGS) have become an alternative approach to improve CPS placement accuracy. These systems use high-resolution preoperative computed tomography (CT) data to create three-dimensional reconstructions of individual vertebrae, which are used to design and manufacture custom-fitted guides that fit the remaining bony surfaces exactly. Predetermined screw entry points and trajectories are then transferred directly to the operative field without the need for intraoperative registration or fluoroscopic interpretation [11-13]. A recent large clinical series and single-centre study in the field of primary cervical surgery has reported high CPS placement accuracy using TGS, with major perforation rates generally reported between 0% and 6% and low rates of revision for screw malposition [14].

However, evidence on the use of TGS specifically in revision cervical spine surgery is still lacking, and the altered anatomy of revision cases presents a certain set of unique challenges that may affect guide fit, the feasibility of the trajectory, and the overall accuracy [15]. This study aimed to assess the accuracy of CPS placement using a patient-specific TGS in the context of revision cervical spine surgery. The secondary aim was to characterise radiographic and screw-related morphometric parameters throughout cervical levels in this revision population, to better contextualise anatomical feasibility, optimise screw trajectory planning, and provide information on safe application of template-guided CPS fixation in this particularly challenging clinical scenario.

## Materials and methods

Study design and patient selection

This was a retrospective observational study conducted at Hokkaido Orthopaedic Memorial Hospital, Sapporo, Japan. The study was approved by the Ethics Committee of Hokkaido Orthopaedic Memorial Hospital (approval number: 113), and all procedures were carried out in accordance with institutional guidelines.

Fifteen consecutive patients who underwent posterior cervical instrumentation using a patient-specific template guide system following prior posterior cervical decompression surgery were included. All patients had experienced previous laminoplasty or laminectomy, and revision cervical spine surgery was required for progressive myelopathy, instability, deformity, or trauma. Patients were included if CT imaging was available postoperatively for evaluation of cervical pedicle screw placement. Patients with incomplete imaging after surgery and insufficient clinical records were excluded.

Preoperative imaging and surgical planning

All patients underwent the preoperative thin-slice CT scanning of the cervical spine with a slice thickness of 1.0 mm or less. CT data were used to create three-dimensional reconstructions of each cervical vertebra C2-C7. According to these reconstructions, the optimal entry points as well as trajectories, diameters, and lengths of the screws were calculated for each pedicle using dedicated preoperative planning software. Planned trajectories of the screws were visualised in sagittal, axial, and coronal planes, and allowed detailed assessment of pedicle morphology and orientation of the screws before surgery.

**Figure 1:**
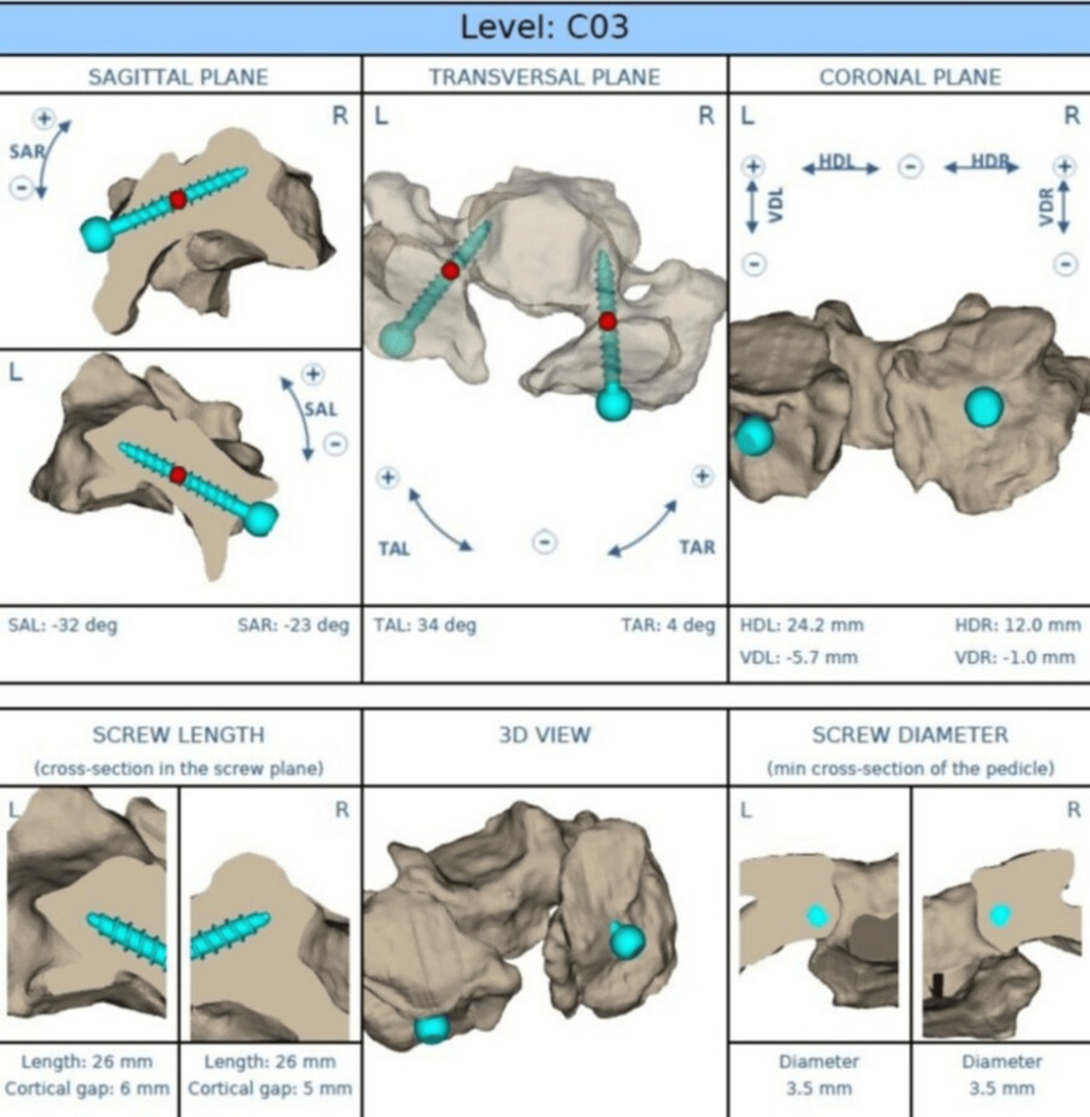
Preoperative planning for pedicle screw placement using the MySpine Cervical® system

Figure 1 shows the workflow of preoperative planning of cervical pedicle screw placement using the MySpine Cervical® system (Medacta international, Castel San Pietro, Switzerland) [14]. Three-dimensional reconstruction of the cervical vertebra was performed by using data from the thin-slice computed tomography to determine the optimal entry point, trajectory, diameter, and length of the screws. The planned trajectory of the screws was visualized in the sagittal, transverse, and coronal planes, which allowed for accurate assessment of the morphology and the orientation of the pedicle before the surgery.

Template guide design

**Figure 2:**
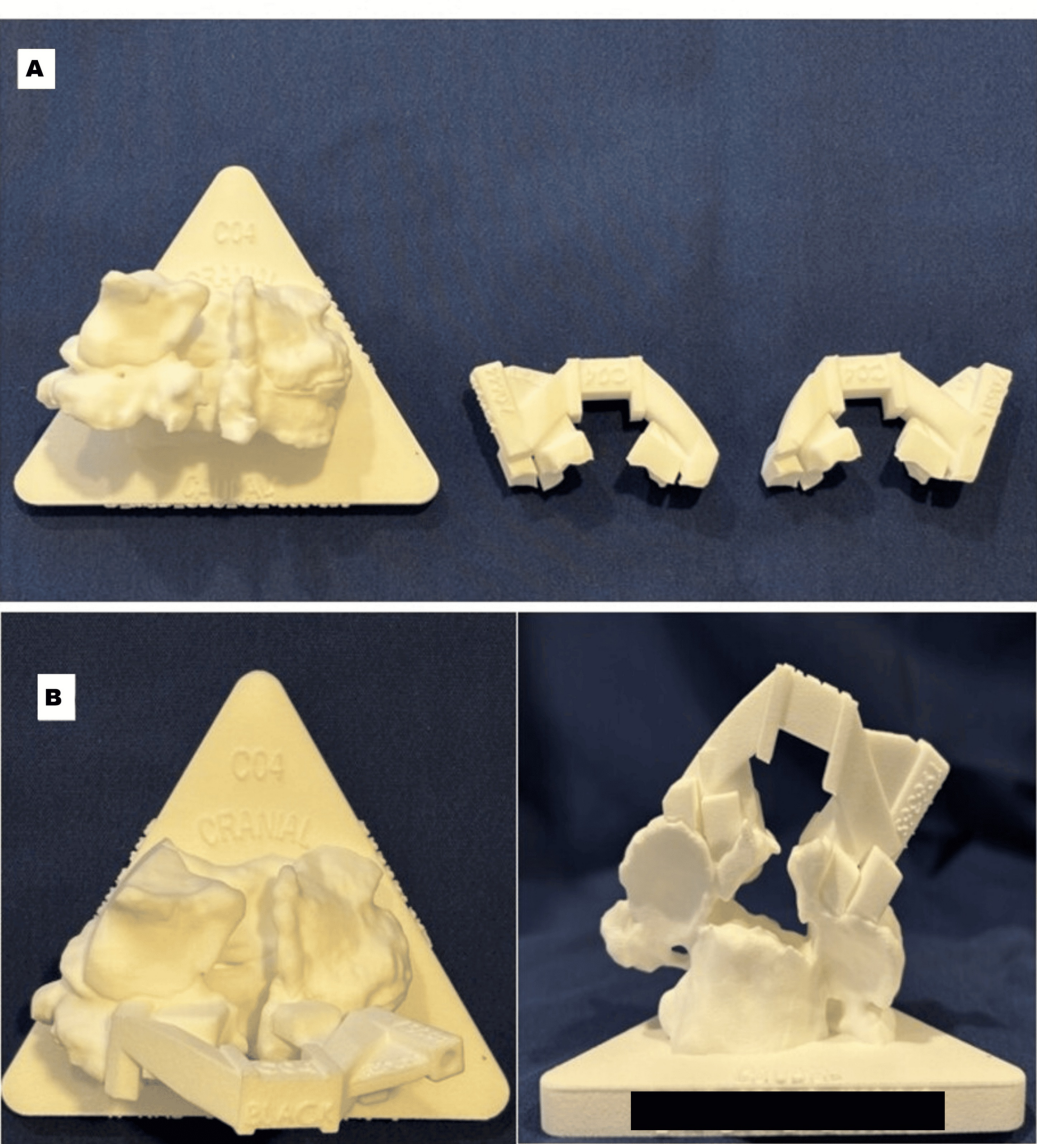
(A) Bone model of the cervical vertebra and patient-specific template guide system for revision surgery; (B) Template guide fitted on the bone model

Patient-specific template guides were created to fit the available bony anatomy of the target vertebra, posteriorly, perfectly. In cases of revision, guide design was performed for partial deficiency of the lamina or lateral mass due to previous decompression procedures. The guides were made to obtain stable seating on the remaining bony surfaces without touching the scarred dura or the neural structures to allow easy transfer of the preoperatively planned screw trajectory to the operative field. The design and application of the patient-specific template guide system for revision cervical spine surgery is shown in Figure 2.

Figure 2A shows the template guide system in a revision surgery setting and the reconstruction model of the bone of a cervical vertebra after a previous posterior decompression procedure with lamina or lateral mass partial defects. Figure 2B shows the corresponding patient-specific template guide to be used to accommodate the altered bony anatomy. The template guide fitted onto the revised model of the bone and shows stable guide positioning despite the lack of any normal anatomical landmarks. Together, these images provide an emphasis on the feasibility of using a patient-specific template guide system in accurate cervical pedicle screw placement during revision surgery.

Surgical technique

A standard cervical midline posterior approach was used in all procedures. Dissection of the soft tissues was done carefully to expose the posterior elements without damaging the bony landmarks necessary to position the patient-specific template guide correctly. The template guide was then placed over the back of the target vertebra and reflected on to ensure it was sitting correctly and in a stable position over the bony anatomy.

According to the preoperative plan, pilot holes were drilled using the template guide sleeves to a predetermined depth of 12 mm. Probing and tapping were also performed through the guide sleeves to establish the intended trajectory. Following this, the guide was removed, and cervical pedicle screws were inserted using a free-hand technique along the prepared path. Therefore, the template guide system functioned as a pilot-hole guidance tool rather than a fully guided screw insertion system. Selective intraoperative lateral fluoroscopy was used to confirm the entry point and sagittal alignment [16].

**Figure 3:**
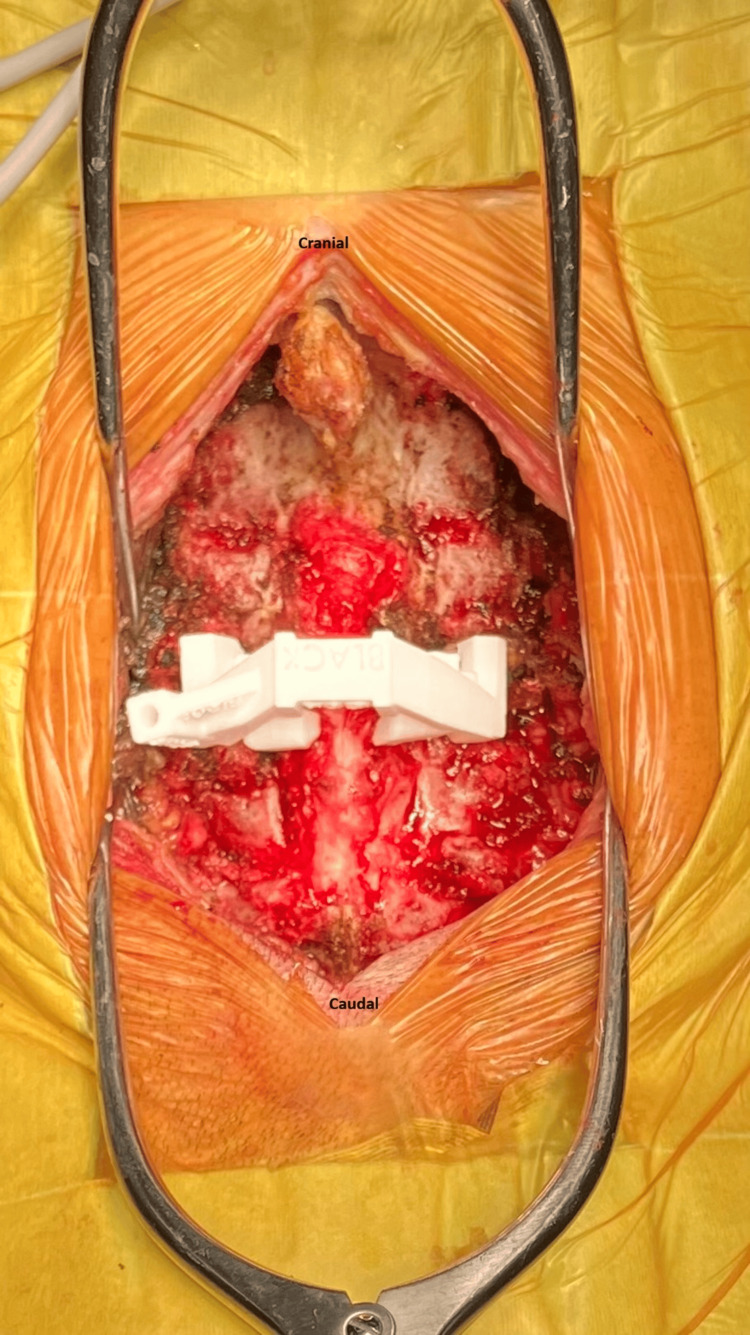
Patient-specific template guide firmly placed on the posterior bony surface

Figure 3 shows the application of the patient-specific template guide in the operation. This guide was clamped on the posterior elements to give a stable fixation during the drilling of the pilot holes to the preset depth, after which probing and tapping through the guide sleeves were done.

Radiographic evaluation and outcome measures

Postoperative CT scans were independently reviewed by two experienced spine surgeons who were not involved in the index procedures. Screw placement accuracy was assessed in all cervical pedicle screws placed from C2 to C7 and was graded according to a modified Gertzbein-Robbins classification, where Grade 0 is a complete containment within the pedicle, grade 1 is deviation lesser than 2 mm, grade 2 is deviation 2-4 mm and grade 3 is deviation more than 4 mm. Grades 0 and 1 were considered clinically acceptable [17].

**Figure 4:**
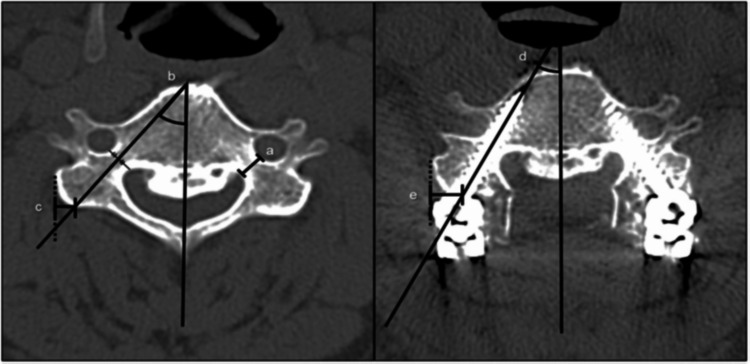
Radiographic parameters of vertebrae and pedicle screw: (a) pedicle width, (b) pedicle transverse angle, (c) pedicle medial offset, (d) pedicle screw transverse angle, (e) pedicle screw medial offset

In addition to the assessment of accuracy, radiographic morphometric parameters were measured on the postoperative CT images. Vertebral parameters included pedicle width, pedicle transverse angle, and pedicle medial offset, and screw-related parameters included pedicle screw diameter, screw length, pedicle screw transverse angle, and pedicle screw medial offset. Measurements were taken in axial and reconstructed planes using standardised measuring techniques. Figure 4 shows the radiographic parameters measured for evaluation of the morphology of the vertebrae and the pedicle screws. Vertebral parameters included pedicle width, pedicle transverse angle, and pedicle medial offset, and screw-related parameters included pedicle screw transverse angle and pedicle screw medial offset. These measurements were made from the postoperative CT scans and were used as the basis for morphometric and comparative analyses among cervical levels.

Statistical analysis

Descriptive statistics were used to summarise the demographics of the patients, surgical characteristics, and pedicle screw distribution. Continuous variables are reported as mean values ± SD, and categorical variables are reported as frequency and percentage. Because the statistical distribution of radiographic parameters was not normal, the interlevel comparisons between cervical vertebral levels (C2-C7) were conducted with the Kruskal-Wallis test. When a statistically significant overall difference was found, post hoc pairwise comparisons were performed using Dunn's test with the use of Bonferroni's correction to control for multiple comparisons. A two-sided p-value of < 0.05 was statistically significant. All statistical analyses were conducted with IBM SPSS Statistics for Windows, version 29.0 (IBM Corp., Armonk, New York, United States).

## Results

Patient demographics and surgical characteristics

A total of 15 patients who underwent revision posterior cervical spine surgery using a patient-specific template guide system were included in the analysis. The mean patient age was 62.6 ± 14.2 years (range, 41-88 years). The mean operative time was 209.8 ± 60.1 minutes, with a mean estimated blood loss of 84.6 ± 55.6 mL. Mean height, weight, and body mass index were 163.9 ± 11.3 cm, 62.4 ± 15.7 kg, and 23.0 ± 4.4 kg/m², respectively. Detailed demographic data are summarised in Table 1.

**Table 1:** Demographic data of patients included in the study (N=15)

Variable	Mean±SD (Range)
Age (years)	62.60±14.237 (41-88)
Time (minutes)	209.80±60.103 (117-327)
Blood loss (cc)	84.60±55.588 (10-194)
Height (cm)	163.920±11.284 (143.5-180.0)
Weight (Kg)	62.393±15.650 (37.1-96.4)
BMI (Kg/m2)	23.0±4.392 (16.5-32.1)

Table 2 provides the surgical features of the revision cases. Most patients had already undergone laminoplasty with French-door laminoplasty (n=11, 73.3%) as the most prevalent primary decompression surgery, laminectomy (n=3, 20.0%), and open-door laminoplasty (n=1, 6.7%). Recurrent cervical spondylotic myelopathy (CSM) following laminoplasty was the most common cause of revision surgery (n=7, 46.6%). Other signs involved CSM with postoperative kyphosis following laminectomy, dropped head syndrome following laminoplasty, ossification of the anterior longitudinal ligament (OPLL) with or without CSM following laminoplasty, retro-odontoid pseudotumor, and post-laminoplasty trauma.

**Table 2:** Surgical characteristics, diagnoses, fusion levels, and pedicle screw distribution in patients undergoing revision cervical spine surgery (N = 15) CSM: cervical spondylotic myelopathy; OPLL: ossification of the anterior longitudinal ligament

Initial decompression procedure	Frequency (Percentage)
French-door laminoplasty	11 (73.3)
Laminectomy	3 (20.0)
Open-door laminoplasty	1 (6.7)
Cause of Revision Surgery	
CSM after laminoplasty	7 (46.6)
CSM with kyphosis after laminectomy	1 (6.7)
Dropped head syndrome after laminoplasty	1 (6.7)
OPLL	2 (13.3)
OPLL with CSM after laminoplasty	1 (6.7)
Retro-odontoid pseudotumor	1 (6.7)
Trauma after laminoplasty	2 (13.3)
Fusion level	
C2-7	4 (20.0)
C2-T1	2 (13.3)
C2-T2	1 (6.7)
C2-3	1 (6.7)
C3-7	1 (6.7)
C4-6	2 (13.3)
C5-7	1 (6.7)
C5-T4	1 (6.7)
C6-T2	1 (6.7)
O-C2 (occiput–C2 fusion)	1 (6.7)
Number of decompressed segments	
0	7 (46.7)
1	1 (6.7)
2	4 (26.7)
3	1 (6.7)
5	2 (13.3)
Number of fused segments	
1	1 (6.7)
2	4 (26.7)
3	2 (13.3)
4	2 (13.3)
5	3 (20.0)

There was a wide range of fusion levels because revision cervical spine surgery is heterogeneous, with short-segment fusions to long-segment fusions, and occiput-C2 fusion in one patient. There were decompressed segments with a range of 0-5 and fused segments with a range of 1-5 levels. The pedicle screws inserted were between two and 11, depending on the patient. Penetration of the screws was not seen in 11 patients (73.3%), and four patients (26.7%) had one penetrated screw each.

Cervical pedicle screw placement accuracy

A total of 90 cervical pedicle screws, inserted from C2 to C7, were assessed with the use of postoperative CT. Overall, 86 (95.6%) screws were completely contained within the pedicle (Grade 0), and four (4.4%) screws showed Grade 1 deviation values of less than 2 mm. All deviations were clinically acceptable, and no neurovascular and neurological complications related to screws placement occurred. Screw distribution and placement accuracy according to vertebral level are presented in Table 3.

**Table 3:** Placement accuracy and deviation of cervical pedicle screw (C2–C7)

Vertebral level	Total screws	Grade 0, n (%)	Grade 1, n (%)
C2	16	16 (100.0)	0 (0.0)
C3	10	9 (90.0)	1 (10.0)
C4	12	11 (91.7)	1 (8.3)
C5	12	11 (91.7)	1 (8.3)
C6	18	17 (94.4)	1 (5.6)
C7	22	20 (90.9)	2 (9.1)
Total	90	86 (95.6)	4 (4.4)

Pedicle width was significantly different at each cervical vertebral level according to the Kruskal-Wallis test (χ² (5) = 19.08, p = .002). Post-hoc Dunn-Bonferroni analysis showed that the pedicle width at C2 was significantly higher than that at C3 (p = .010), C4 (p = .031), and C6 (p = .048). These findings are summarised in Table 4.

**Table 4:** Comparison of pedicle width across cervical levels (C2–C7)

Analysis	Test statistic	p value
Kruskal–Wallis test		
C2–C7	χ² (5) = 19.08	.002
Post-hoc Dunn–Bonferroni		
C2 vs C3	Z = 3.42	.010
C2 vs C4	Z = 3.08	.031
C2 vs C6	Z = 2.95	.048

Inter-level comparison of radiographic and screw-related parameters

Kruskal-Wallis tests showed significant differences between cervical levels (C2-C7) for all the radiographic and parameters related to the screws, analysed (Table 5). Pedicle width differed significantly across levels, χ² (5, N = 90) = 19.08, p = .002, with post-hoc Dunn-Bonferroni analyses showing that pedicle width at C2 was significantly greater than at C3 (adjusted p = .010), C4 (adjusted p = .031), and C6 (adjusted p = .048). Pedicle transverse angle also varied significantly by level, χ² (5, N = 90) = 36.19, p < .001, with significant differences primarily involving lower cervical levels, particularly C7. Significant inter-level differences were further observed for pedicle medial offset, χ² (5, N = 90) = 53.81, p < .001; pedicle screw diameter, χ² (5, N = 90) = 53.72, p < .001; pedicle screw length, χ² (5, N = 90) = 29.57, p < .001; pedicle screw transverse angle, χ²(5, N = 90) = 59.28, p < .001; and pedicle screw medial offset, χ² (5, N = 90) = 48.92, p < .001. Post-hoc Dunn-Bonferroni analyses indicated that C2 frequently differed significantly from mid- and lower-cervical levels across these parameters. Collectively, these findings show a great deal of anatomical variation in the morphology of the pedicle and the parameters associated with screws, among the cervical levels, and the greatest differences were seen at the upper cervical spine.

**Table 5:** Kruskal–Wallis and post-hoc Dunn–Bonferroni analyses of radiographic parameters across cervical levels (C2–C7) Note: Values were compared across cervical levels (C2–C7) using the Kruskal–Wallis test. Pairwise comparisons were performed using Dunn–Bonferroni post-hoc analysis.

Parameter	χ² (df = 5)	p value	Post-hoc Comparison	p value
Pedicle width (mm)	19.08	.002	C2 vs C3	.010
			C2 vs C4	.031
			C2 vs C6	.048
Pedicle transverse angle (°)	36.19	< .001	C7 vs C6	.018
			C7 vs C4	< .001
			C7 vs C5	< .001
			C2 vs C5	.010
Pedicle medial offset (mm)	53.81	< .001	C2 vs C3	.003
			C2 vs C4	.002
			C2 vs C5	.007
			C2 vs C6	< .001
			C2 vs C7	< .001
			C5 vs C7	.046
			C6 vs C7	.040
Pedicle screw diameter (mm)	53.72	< .001	C5 vs C2	< .001
			C5 vs C7	< .001
			C3 vs C2	< .001
			C3 vs C7	.001
			C4 vs C2	< .001
			C4 vs C7	.001
			C6 vs C2	< .001
			C6 vs C7	.004
Pedicle screw length (mm)	29.57	< .001	C4 vs C2	< .001
			C5 vs C2	.001
			C3 vs C2	.042
Pedicle screw transverse angle (°)	59.28	< .001	C2 vs C3	.001
			C2 vs C4	< .001
			C2 vs C5	< .001
			C2 vs C6	< .001
			C4 vs C7	.001
			C5 vs C7	< .001
Pedicle screw medial offset (mm)	48.92	< .001	C2 vs C3	.047
			C2 vs C4	.002
			C2 vs C5	< .001
			C2 vs C6	< .001
			C2 vs C7	< .001

**Figure 5:**
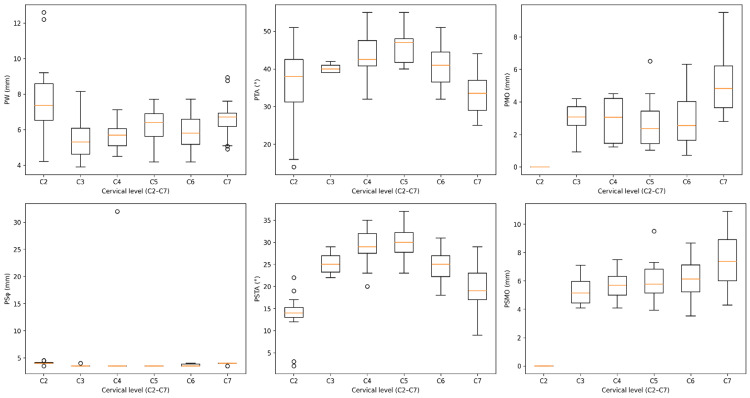
Comparison of radiographic parameters for each cervical vertebral level (C2–C7) and cervical pedicle screws across levels. Inter-level differences were analysed using the Kruskal–Wallis test with Dunn–Bonferroni multiple comparison post-hoc analysis.

Box-and-whisker plots of radiographic and screw-related parameters of each cervical vertebral level (C2-C7) are presented in Figure 5. The distribution of each parameter is presented as median (interquartile range (IQR)), pointing out the inter-level anatomical variation. Significant differences between the cervical levels were determined by the Kruskal-Wallis test with the Dunn-Bonferroni post-hoc test. Upper cervical levels and especially C2 were found to have different morphometric features than mid- and lower-cervical levels, supporting the importance of planning preoperatively to match individual levels.

## Discussion

Revision cervical spine surgery is one of the most technically challenging situations in which to place CPS because of altered posterior anatomy, lost conventional bony landmarks, epidural scarring, and distorted spatial orientation in the context of previous decompression surgery. The present study shows that CPS placement with a patient-specific TGS can be accurately and safely performed clinically in revision settings, with 95.6% of screws being fully contained within the pedicle without major (Grade 2 or 3) perforations. These findings are notable for the fact that all cases were associated with previous posterior cervical surgery, which is a population noted to have a high risk of screw misplacement and neurovascular complications.

Despite improvements in surgical technique and imaging, freehand CPS insertion is still associated with considerable perforation rates in the current series. Mahesh et al. conducted a CT-based evaluation of 577 cervical pedicle screws and reported a total perforation rate of 25.6% with clinically significant Grade 2-3 breaches occurring in more than 8% of cases [18]. Similarly, Hojo et al. showed breach rates close to 15% even with the use of lateral fluoroscopy [19]. These risks are likely to be increased in revision surgery, where standard anatomical references are unreliable or missing.

Navigation-based and robotic-assisted CPS placement techniques have been more widely used to address these risks; however, recent evidence has shown that they are not 100% accurate. The reported perforation rates with O-arm-based navigation were between 3.8 and 22.9% [20,21], while perforation rates of robotic-assisted systems were between 15.9 and 17.3% [22]. Importantly, both navigation and robotic platforms rely on precise intraoperative registration, which could be impaired in revision cases because of multilevel posterior bone defects, altered alignment of the vertebrae, or instability during exposure [14]. In contrast, TGS converts a preoperatively planned trajectory of the screw transfer directly to the operative field without the need for intraoperative registration. This feature is especially useful with revision surgery, where distorted anatomy can hamper the accuracy of navigation-based systems.

Fujita et al. reported a perforation rate of 1.3% with no major breaks using TGS [23], while Farshad et al. reported a clinically acceptable placement in more than 95% of screws [24]. A systematic review by Mahmoud et al. confirmed that template-guided CPS placement is as accurate or more accurate than navigation-assisted techniques [21].In our study, no screw-related complications, including neurovascular or spinal cord injury, were observed, and all deviations were minor without clinical sequelae.

The most recent and largest evaluation of the use of TGS-assisted CPS placement was published by Fukushima et al. in 2025, analysing 437 screws with a major perforation rate of only 0.5% [14]. Notably, in their cohort, they had 11 cases of revision, for which the perforation rate was low, and all the deviations were minor. The present study builds on these results but focuses on revision cervical spine surgery only, and shows similarly high accuracy, suggesting that the benefits of TGS are maintained even in anatomically compromised revision settings.

Revision surgery creates peculiar problems not completely covered in studies overwhelmingly led by primary cases. Prior laminoplasty or laminectomy often leads to partial or complete loss of the lamina, spinous process, or lateral mass, which decreases the surface area for guide seating and leads to a higher risk of intraoperative guide instability [14]. In the current study, the TGS was shaped especially to follow the rest of the posteriorly oriented bony anatomy, without contact between the scarified dura or neural tissue. This customised design probably played a major role in the lack of significant screw deviations and neurovascular complications.

Fukushima et al. similarly emphasised careful template-bone matching and ensuring that the safety margin from the dura is adequate as critical factors to help achieve high accuracy in revision cases [14]. Together, these results indicate that careful preoperative planning and guide designs are important factors in the successful application of TGS in revision cervical spine surgery.

The observed significant inter-level differences in pedicle width, transverse angle, medial offset, and parameters related to the screws are consistent with earlier morphometric studies based on CT of the cervical spine [14]. In particular, the increased width and different morphology at the level of C2 from subaxial levels emphasise the need for planning by level and not to rely on standardised planning trajectories. Small pedicle width, particularly at C3 and C4, has recently been found to be a significant risk factor for perforation of the CPS, even when TGS is used [25].

In line with earlier reports, the premeditated screw paths in the current study demonstrated reduced transverse angles and greater intermediary offsets relative to native pedicle anatomy [26]. While such modifications have been proposed to lower the risk of lateral pedicle wall breach and vertebral artery injury, this was not directly evaluated in the present study.

In revision cases, posterior anatomical landmarks, including the spinous process, lamina, and facet joints, may be altered or absent due to prior decompression, complicating free-hand screw placement. Nevertheless, the lateral mass notch is often preserved and may serve as a reliable reference for determining the entry point and trajectory of cervical pedicle screws.

Although our findings demonstrate a high rate of accurate screw placement, comparisons with navigation and robotic systems are indirect and based on previously published data. As this study did not include a control or comparator group, no conclusions regarding superiority or equivalence can be drawn. Further prospective comparative studies are required to validate these findings.

Clinical implications

From the clinical point of view, the results of this study advocate the use of patient-specific TGS as a helpful and reliable adjunct for the placement of CPS in revision cervical spine surgery. The lack of Grade 2-3 breach and neurovascular complications is of special significance because of the high baseline risk associated with revision procedures. Compared with navigation and robotic systems, TGS has several practical benefits, including the avoidance of registration errors, reduced reliance on intraoperative imaging, and the consistency of transfer of preplanned trajectories to the surgical field.

Additionally, recent evidence suggests TGS may decrease the learning curve associated with CPS placement, although experience of the surgeon is still an important determinant of accuracy [27]. In the realm of revision settings, an anatomical complexity exists, for which TGS seems to offer an added margin of safety for both experienced and less experienced surgeons.

Limitations and recommendations

This study had a few limitations. It was retrospective in design, was a single-center study, and had a relatively small sample size. The lack of a direct comparator group of individuals using navigation or robotic systems makes it impossible to draw definitive conclusions on superiority. Furthermore, long-term clinical outcomes, fusion rates, and functional measures were not evaluated. Future multicentre studies that focus specifically on revision cervical spine surgery are required to validate these observations and to further determine patient and anatomy-specific risk factors for CPS deviation.

## Conclusions

The present study demonstrates that patient-specific TGS allows for highly accurate and safe placement of CPS in revision cervical spine surgery with an accuracy comparable to or greater than that reported for navigation and robotic systems, while avoiding their limitations in anatomically distorted revision cases. These results support the expanded use of TGS as a useful tool in posterior cervical fixation in complex revision scenarios.
